# Elevated Serum Polybrominated Diphenyl Ethers and Alteration of Thyroid Hormones in Children from Guiyu, China

**DOI:** 10.1371/journal.pone.0113699

**Published:** 2014-11-21

**Authors:** Xijin Xu, Junxiao Liu, Xiang Zeng, Fangfang Lu, Aimin Chen, Xia Huo

**Affiliations:** 1 Laboratory of Environmental Medicine and Developmental Toxicology, and Provincial Key Laboratory of Infectious Diseases and Molecular Immunopathology, Shantou University Medical College, Shantou, China; 2 Department of Cell Biology and Genetics, Shantou University Medical College, Shantou, China; 3 Department of Epidemiology, University Medical Center Groningen, University of Groningen, Groningen, the Netherlands; 4 Division of Epidemiology and Biostatistics, Department of Environmental Health, University of Cincinnati College of Medicine, Cincinnati, United States of America; Institute for Health & the Environment, United States of America

## Abstract

Informal electronic waste (e-waste) recycling results in serious environmental pollution of polybrominated diphenyl ethers (PBDEs) and heavy metals. This study explored whether there is an association between PBDEs, heavy metal and key growth- and development-related hormones in children from Guiyu, an e-waste area in southern China. We quantified eight PBDE congeners using gas chromatographic mass spectrometry, lead and cadmium utilizing graphite furnace atomic absorption spectrometry, three thyroids with radioimmunoassay and two types of growth hormones by an enzyme-linked immune-sorbent assay (ELISA) in 162 children, 4 to 6 years old, from Guiyu. In blood, median total PBDE was 189.99 ng/g lipid. Lead and cadmium concentrations in blood averaged 14.53±4.85 µg dL^−1^ and 0.77±0.35 µg L^−1^, respectively. Spearman partial correlation analysis illustrated that lead was positively correlated with BDE153 and BDE183. Thyroid-stimulating hormone (TSH) was positively correlated with almost all PBDE congeners and negatively correlated with insulin-like growth factor binding protein-3 (IGFBP-3), whereas free triiodothyronine (FT3) and free thyroxine (FT4) were negatively correlated with BDE154. However, no correlation between the hormones and blood lead or cadmium levels was found in this study. Adjusted multiple linear regression analysis showed that total PBDEs was negatively associated with FT3 and positively associated with TSH. Notably, FT4 was positively correlated with FT3, house functions as a workshop, and father's work involved in e-waste recycling and negatively correlated with vitamin consumptions. TSH was negatively related with FT4, paternal residence time in Guiyu, working hours of mother, and child bean products intake. IGFBP-3 was positively correlated with IGF-1 and house close to an e-waste dump. These results suggest that elevated PBDEs and heavy metals related to e-waste in Guiyu may be important risk factors for hormone alterations in children.

## Introduction

Electronic waste (e-waste) is an emerging environmental health issue because of the explosive growth of electronic products and its rapid update rate and accumulation worldwide, as well as inadequate recycling technology and informal recycling activities [1]. Guiyu, a town with a total area of 52 km^2^ and a population of 139,000 (2010) located in Shantou, China, is one of the largest e-waste destinations in the world. More than 6,000 small-scale family-run workshops and nearly 160,000 workers (including approximately 100,000 migrant farmer laborers) are involved in the business of e-waste dismantling and recycling with crude and uncontrolled methods that extensively introduce environmental pollutants into the community [2]–[5].

Polybrominated diphenyl ethers (PBDEs) are one of the most common used materials among the organic contaminants in electronic equipment, which are a large group of brominated compounds widely utilized as flame retardant additives in the products of commodity such as automobiles, airplanes and furniture [6]. Compared with other sites from Nigeria or China, higher levels of PBDE are found in soil, air, sediment, as well as freshwater fish collected from Guiyu [7], [8]. PBDE concentrations in umbilical cord blood are also higher than those from nearby reference areas without e-waste recycling activities [4], [9]. The metabolites and environmental derivatives of PBDE have a similar chemical structure as thyroid hormones (TH), especially for thyroxine (T4) [10], [11]. A study shows that PBDEs may mimic or compete with TH to disrupt the function of target organs [12]. In addition, epidemiological studies suggest that occupational and environmental exposure to persistent organic pollutants (POPs) might alter insulin-like growth factor-1 (IGF-1) and insulin-like growth factor-binding protein-3 (IGFBP-3) levels or their gene expression, which are involved in the insulin-like growth factor (IGF) axis when impact on growth and development [4], [13]–[15].

Guiyu is a well-known e-waste disassembling town mainly polluted by metal chemicals and persistent organic pollutants (POPs). Our previous studies found that heavy metals, such as lead and cadmium, in Guiyu children are much higher than that in nearby regions Chendian and Chaonan, which are also located in Shantou, China. It is notable that distance from Guiyu to Chendian or Chaoan is less than 40 kilometers. Inhabitants who live in the three regions mentioned above have similar life-styles except that people living in Guiyu are engaged in activities related to e-waste recycling. The percentages of blood lead concentration exceeding 10 µg/dL in Guiyu children were 81.83% (2004), 70.8% (2006), 69.9% (2008), and 88.02% (2010), from 2004 to 2010, respectively [2], [16], [17]. Cadmium levels in human and environmental samples from Guiyu are also higher than those in other places without e-waste recycling [7], [18]–[21]. Similar to the case of PBDEs, lead and cadmium may also relate to hormone alteration. In adolescents, long-term low-level lead exposure may reduce FT4 levels [22]; while a decrease in TSH levels was correlated with blood lead levels in women [23]. In addition, animal studies show that exposure to 25 or 50 mg/L cadmium chloride (CdCl_2_) can increase median TSH levels in rats [24]. Kortenkamp et al. reported that cadmium, and some other heavy metals should be regarded as estrogen mimics [25]. This endocrine-disruption of thyroid function might have significant impact on growth and development during the rapid growth stages of the central nervous system of children, as children are the most vulnerable to the harmful effects of environmental insults [26], [27].

However, few epidemiology studies have reported an association among PBDEs and heavy metal (lead and cadmium) co-exposure with thyroid and growth hormones in children, especially from an e-waste recycling area. This study was designed to evaluate the relationships between these co-exposures and hormone levels among children living in Guiyu.

## Materials and Methods

### Participants

Children greater than 6- or less than 4-years old was precluded before blood collection. According to the exclusion principle, 167 children, 4-to-6-years of age, were recruited from a kindergarten of Guiyu in October, 2010. The study protocol was approved by the Human Ethics Committee of Shantou University Medical College. Parents or guardians received detailed explanations of the study and potential consequences prior to enrollment and gave their written informed consent. They were interviewed by well-trained research staff using questionnaires involving information covering the dwellings (residence close to an e-waste dismantling site, passive smoking), child behavior (frequency of hand-to-mouth contact), diet and nutritional conditions, parent education, occupation, and social status. Parent education was classified by their relationship to e-waste recycling activities (e.g. unrelated, transporting e-waste, sorting e-waste, splitting e-waste, acid baths, and burning to recover metals). In addition, child physical growth and development indices, such as body height, weight, and head and chest circumferences were measured at the time of blood sample collection.

### Blood sample collection

The children were required to fast after dinner until the blood samples were collected on the following morning. For each child, 0.5 mL whole blood with heparin and 5 mL blood without anticoagulant were obtained by trained nurses. After coagulation, serum samples were instantly separated into 2 mL and 0.5 mL acetone-washed clean glass containers, numbered, immediately transported to the laboratory, and stored at −80°C. In addition, whole blood was stored at −20°C as soon as possible.

### Data collection

#### PBDE analysis

PBDE concentrations of 145 children in 167 were measured due to the limited volume of blood samples. PBDE congeners such as BDE28, 47, 99, 100, 153, 154, 183, and 209 were identified. The standard mixture containing eight congeners was purchased from Cambridge Isotope Laboratories (CIL). Serum PBDE concentrations were analyzed based on a previously published method, with minor modifications [28]. The serum sample was first extracted with formic acid and acetonitrile, and equilibrated in an ultrasonic bath, then loaded on the solid-phase extraction cartridge (Plexa) and washed with water (Milli-Q), dried and subsequently eluted with dichloromethane. The eluate of PBDE was concentrated under a gentle stream of nitrogen to 1 mL, then applied to a silica gel/sulfuric acid column, and eluted with dichloromethane. After that, the eluate was concentrated to 50 µL and transferred to glass inserts in amber GC vials for gas chromatography/mass spectrometry (GC/MS) analysis. The final samples were analyzed with an Agilent 7890A-5975C GC/MS (Agilent Technologies, America), with electro-ionization used in the selected ion monitoring mode. Concentrations of PBDEs in individual samples were reported as ng/g lipid weight. Total cholesterol (CHOL) and triglycerides (TG) were determined enzymatically in a separate aliquot of serum by a clinical laboratory. Total lipid (TL) was calculated according to the following formula: TL (g/L)  = 1.12×CHOL+1.33×TG+1.48 [29].

#### Thyroid hormone analysis

Serum free T3 (FT3) and free T4 (FT4) concentrations were quantified in duplicate by radioimmunoassay, using human serum T3 and T4 standard substance, respectively (Chemclin, Biotechnology Corp., Beijing, China). The sensitivities of the assays of FT3 and FT4 were 0.5 pmol/L. Serum thyroid-stimulating hormone (TSH) concentrations were measured by ultrasensitive third-generation immunochemiluminometric assay (Advia Centaur CP, Siemens, German), and the limit of detection of TSH was 0.01 µIU/mL.

#### Growth hormone levels

Insulin-like growth factor-1 (IGF-1) and insulin-like growth factor binding protein-3 (IGFBP-3) in serum were examined by enzyme-linked immune-sorbent assay (ELISA).

#### Lead and cadmium levels

Lead and cadmium in whole blood were determined by graphite furnace atomic absorption spectrometry (GFAAS, ZEEnit 650, Germany) using an auto sampler (MPE60) with an injection volume set at 10 µL. The detection methods were based on previously published papers [21].

### Statistical analysis

Normality was assessed by quantile-quantile plots and Kolmogorov-Smirnov tests for all continuous variables. The two independent sample t-tests were utilized to compare two sets of normally distributed data. The Mann–Whitney U test was applied to the two sampling site comparison because of the non-normal distribution. The One-Way ANOVA was available to the comparison of multiple sets of data normally distributed. The Pearson Chi-square test was used as a comparison between two or more groups between ranked characters or subjects. PBDE levels below the detection limit were defined as zero [30], [31]. Covariates, such as gender, age, and BMI that have been reported for associations with thyroid hormones elsewhere [32], were adjusted in multivariate models. Spearman rank correlation analysis and multiple stepwise regression analysis were used to examine relationships between PBDEs, lead, cadmium, TSH, FT3, FT4, IGF-1, IGFBP-3 and factors in the questionnaire. All statistical tests were two-sided with a significance level of 0.05. Statistical analyses were conducted using SPSS version 13.0 (SPSS Inc., Chicago, IL, 2004).

## Results

### PBDE levels in children

Concentrations of PBDE were expressed on a serum lipid basis. The frequencies of detection of each PBDE were all greater than 95% except for BDE209, which was 71.72%. The levels and distribution for the eight PBDE congeners and their sums are shown in Table 1 and Table 2. First, the geometric mean (95% CI) of the total PBDE was 162.98 (141.25–186.21) ng/g lipid. Besides, total PBDE concentration was 582.35 ng/g lipid in this study. In addition, the concentration of total PBDE exceeded 100 ng/g lipid for 79% of the children (115/145) recruited from Guiyu. The highest concentrations of PBDE congener were BDE209, which ranged from undetectable to 470.29 ng/g lipid, with a geometric mean value of 184.19 ng/g lipid. In particular, BDE209 contributed to 70% of the total PBDE burden. The second most abundant PBDE congener was BDE153, with concentrations varying from undetectable to 94.39 ng/g lipid. BDE153 accounted for approximately 20% of the total PBDE burden and ranked only second to BDE209. In addition, we found high concentrations of BDE47 and BDE28, which represent 9% and 8% of the total PBDE burden excluding BDE209, respectively. It is worth mentioning that BDE153 increased with age (r = 0.214; *P*<0.05), while BDE154 decreased with BMI (r = −0.169; *P*<0.05). No significant difference in any of the eight PBDEs or the total PBDE was found between boys and girls (*P*>0.05). In addition, the other PBDE congener and total PBDE concentrations was not altered by age (all *P*>0.05).

**Table 1:** Study Subjects Characteristics.

Characteristics	Total (162)	Male (86)	Female (76)	*p* value
Age (years)	4.87±0.66	4.85±0.72	4.89±0.60	0.749^ξ^
Height (cm)	105.5±6.27	105.10±5.77	105.00±2.08	0.927^ξ^
Weight (kg)	17.06±2.53	17.43±2.83	16.64±2.08	0.045^ξ^
Head circumference (cm)	49.22±1.43	49.64±1.38	48.75±1.35	0.000^ξ^
Chest circumference (cm)	50.50(50.00–52.00)	51.00(50.00–52.00)	50.00(50.00–52.00)	0.036^δ^
BMI (kg/m^2^)	15.42±1.47	15.71±1.52	15.09±1.34	0.008^ξ^
Heavy metals exposure				
Lead (µg/dL)	14.53±4.85	15.05±4.63	13.94±5.06	0.148^ξ^
Cadmium (µg/L)	0.77±0.35	0.81±0.42	0.73±0.25	0.121^ξ^
PBDEs exposure (ng/g lipid)				
BDE28	4.55(3.27–14.37)	4.55(2.94–12.62)	4.55(3.63–14.65)	0.036^δ^
BDE47	4.66(2.56–13.61)	4.66(2.53–16.29)	4.66(2.57–12.16)	0.290^δ^
BDE99	4.01(2.32–8.92)	4.00(2.30–11.26)	4.01(2.33–6.90)	0.324^δ^
BDE100	2.84(2.21–4.14)	2.84(2.21–4.43)	2.84(2.22–3.72)	0.727^δ^
BDE153	12.03(7.79–19.37)	12.03(7.99–21.84)	12.03(7.59–17.84)	0.405^δ^
BDE154	3.99(3.12–5.69)	3.99(3.31–6.79)	3.99(3.02–4.97)	0.234^δ^
BDE183	6.06(4.65–9.19)	6.06(4.65–8.37)	6.06(4.53–9.83)	0.914^δ^
BDE209	146.10(0.00–196.41)	146.10(0.00–193.77)	146.10(111.65–200.99)	0.409^δ^
ΣPBDE	189.99(148.54–288.41)	184.24(135.63–274.46)	197.27(157.25–295.61)	0.489^δ^
Hormones				
FT3 (pmol/L)	6.28±1.44	6.29±1.50	6.28±1.38	0.958^ξ^
FT4 (pmol/L)	17.78±4.87	18.47±4.74	16.99±4.92	0.052^ξ^
TSH (µIU/mL)	2.85±1.09	2.85±1.08	2.86±1.10	0.978^ξ^
IGF-1 (ng/mL)	510.79±254.84	519.77±230.41	500.62±281.15	0.635^ξ^
IGFBP-3 (ng/mL)	60.97±29.20	62.51±28.74	59.22±29.79	0.476^ξ^

Mean values with Mean±SD, Median values with interquartile range, Ratio value with n (%), Guiyu, China, 2010.

Abbreviations: BDE, brominated diphenyl ether; BMI, body mass index.

*ξ*: T-test.

*δ*: U-test.

**Table 2:** PBDEs (ng/g lipids), Thyroid Hormone and Growth Hormone in Serum of Children, Guiyu, China, 2010.

Congener	Detection Frequency (%)	GM	95% CI	Min	Max	Percentile				
						10th	25th	50th	75th	90th
PBDEs (n = 162)										
BDE28	97.93	7.10	6.03–8.32	ND	96.02	2.51	3.02	4.71	17.02	34.63
BDE47	90.34	7.92	6.46–9.77	ND	97.56	1.51	2.48	4.93	21.37	50.07
BDE99	97.24	5.22	4.47–6.17	ND	63.09	1.67	2.22	4.27	9.88	19.70
BDE100	100.00	3.33	3.02–3.63	1.36	45.17	1.85	2.16	2.85	4.38	6.63
BDE153	97.93	13.17	11.75–14.79	ND	94.39	4.72	7.60	12.28	21.78	37.42
BDE154	100.00	4.66	4.27–5.13	1.81	54.60	2.48	3.00	3.99	6.48	10.54
BDE183	100.00	6.88	6.17–7.59	2.53	70.97	3.33	4.37	6.04	9.59	15.34
BDE209	71.72	184.19	173.78–199.53	ND	470.29	ND	ND	146.10	202.69	280.69
ΣPBDE				16.56	582.35	30.74	135.68	208.08	301.57	396.06
Hormones (n = 162)										
FT3 (pmol/L)	100.00	6.11	5.89–6.31	2.22	10.00	4.33	5.31	6.13	7.04	7.93
FT4 (pmol/L)	100.00	17.10	16.22–17.78	7.96	35.21	11.15	14.17	17.33	21.00	23.64
TSH (µIU/mL)	100.00	2.66	2.51–2.82	0.97	6.67	1.59	2.12	2.64	3.43	4.64
IGF-1 (ng/mL)	96.55	450.57	416.87–489.78	66.81	1641.62	216.02	306.14	491.63	660.67	886.88
IGFBP-3 (ng/mL)	97.93	52.80	47.86–57.54	8.49	111.24	23.77	32.84	53.78	92.07	99.14
Heavy metals (n = 162)										
Lead (µg/dL)	100.00	13.88	13.18–14.45	7.81	35.39	9.82	11.24	13.40	16.40	20.50
Cadmium (µg/L)	100.00	0.72	0.68–0.76	0.27	3.51	0.47	0.54	0.73	0.90	1.18

Abbreviations: CI, confidence interval; BDE, brominated diphenyl ether; FT3, free triiodothyronine; FT4, free thyroxine; IGF-1, insulin-like growth factor 1; IGFBP-3, insulin-like growth factor binding protein 3; ND, not detectable; TSH, thyroid-stimulating hormone.

### Blood lead and cadmium levels

Table 1 show the blood lead and cadmium levels of children from Guiyu. The mean concentrations of lead and cadmium were 14.53±4.85 µg/dL, and 0.77±0.35 µg/L, respectively. Among children from Guiyu in this study, all of them could be identified with elevated blood lead levels (≥5 µg/dL) according to the U.S. CDC, 2012 reference criterion [33]. The proportion of children with blood lead levels greater than 10 µg/dL was 88.0%, with 10.2% (147/167) having blood lead levels higher than 20.0 µg/dL. In addition, the blood cadmium levels observed in this study exceeded the current range of normal values (≤0.2 µg/L) [34], [35]. However, no different of blood lead and cadmium were found between male and female.

### Thyroid hormones and growth hormones

Average serum concentrations of FT3 and FT4 were 6.28±1.44 pmol/L and 17.78±4.87 pmol/L, respectively. The mean concentrations of TSH, IGF-1, and IGFBP-3 were 2.85±1.09 µIU/mL, 510.79±254.84 ng/mL, and 60.97±29.20 ng/mL, respectively (Table 1). Among them, one participant had low FT3 (<3.2 pmol/L) and another one had low FT4 (<8.56 pmol/L), whereas none had low TSH (<0.35 µIU/mL). In addition, two children had high FT3 (>9.3 pmol/L), 7 other children had high FT4 (>25.8 pmol/L), and two had elevated TSH concentrations (>5.5 µIU/mL) based on reference ranges. Moreover, FT3 had a positive association with BMI (*P*<0.05), whereas IGFBP-3 had a negative association with age in a linear fashion (*P*<0.05). Furthermore, there is no association between FT3, FT4, TSH, IGF-1 and age in this study. Compared with girls, boys had higher levels of FT4.

### Association between PBDEs and related factors in children

Relationship between PBDEs and related factors were analyzed with the spearman rank correlation analysis (Table 3 and Table S1). Dietary choosiness had a positive correlation with BDE28. Frequency of eating dairy and frequency of calcium consumption had a positive correlation with BDE47. Frequency of calcium consumption also had a positive correlation with BDE99. Residence of the child had a positive correlation with BDE100. Age and residence of child had a positive correlation with BDE153, whereas frequency of eating dairy had a negative correlation with BDE153. BMI, dietary choosiness, and residence of child had a positive correlation with BDE154, while frequency of eating dairy had a negative correlation with BDE154. Extent of passive smoking and house close to an e-waste recycling site had positive correlations with BDE183. There was no correlation between BDE209, total PBDE and related factors found in this study. Multiple stepwise regression analysis was used to evaluate factors related to each congener of PBDEs in children from Guiyu. TSH was positively correlated with BDE28, BDE47, BDE99, BDE209, and total PBDE. FT3 was negatively correlated with BDE99, BDE209, and total PBDE. IGFBP-3 was negatively correlated with BDE47. Biting fingers was positively correlated with BDE28 and BDE47. Eating dairy was negatively correlated with BDE154 and BDE209. Bean products intake was negatively correlated with BDE28 and BDE100. Blood lead positively correlated with BDE153.

**Table 3:** Spearman correlation coefficients between PBDEs and some investigated factors in children, Guiyu, China, 2010.

Related Factors	BDE28	BDE47	BDE100	BDE99	BDE154	BDE153	BDE183	BDE209	*Σ*PBDE
Age (years)	−0.018	−0.102.	−0.049	−0.038	0.115	0.204*	0.058	0.060	0.033
BMI	0.094	0.088	−0.053	−0.066	0.169*	−0.139	−0.120	−0.098	0.016
The frequency of eating dairy	−0.001	0.177*	−0.102	−0.001	−0.218*	−0.177*	−0.154	0.120	−0.039
The frequency of taking calcium tablet	0.104	0.203*	0.125	0.174*	0.066	0.047	0.017	−0.060	−0.034
The frequency of passive smoking	0.134	0.113	0.138	−0.017	0.147	0.144	0.187*	0.069	0.091
House close to e-waste	0.133	0.126	0.150	0.012	0.132	0.092	0.192*	−0.061	0.030
The habit of dietary choosiness	0.187*	0.051	0.162	0.109	0.174*	0.068	0.089	0.063	0.102
Residence of the child	0.135	0.011	0.169*	0.095	0.180*	0.189*	0.121	0.053	0.089

Abbreviations: BDE, brominated diphenyl ether; BMI, body mass index.

*P<0.05, **P<0.01.

### Association between heavy metals and related factors in children

Relationship between heavy metals and related factors were analyzed with the spearman rank correlation analysis (Table 4). Gender as male and working hours of the mother had a positive correlation with blood lead. Weight had a negative correlation with blood lead. Head circumference and chest circumference had a positive correlation with blood cadmium. Children suffered from any disease and mother's education levels had a negative correlation with blood cadmium.

**Table 4:** Spearman correlation coefficients between blood heavy metals and some investigated factors in children, Guiyu, China, 2010.

Related factors	Lead(n = 162)		Cadmium(n = 162)	
	*r_s_*	*p*	*r_s_*	*p*
Gender	−0.190	0.016	−0.072	0.364
Head circumference (cm)	0.153	0.054	0.268	0.001
Chest circumference (cm)	0.126	0.111	0.233	0.003
Birth weight	−0.239	0.013	0.010	0.916
Whether suffered from any disease	0.013	0.885	−0.190	0.027
Mother's education levels	−0.073	0.399	−0.170	0.046
Working hours of the mother	0.181	0.034	0.066	0.444

### Associations between hormones and related factors in children

Relationship between hormones and risk factors were analyzed with the spearman rank correlation analysis (Table 5). BMI was negatively correlated with FT3. Gender as female, vitamin consumption, suffered from any diseases, drinking well water, working hours of mother, and house functions as a workshop were negatively correlated with FT4. Biting fingers, frequency of bean products intake, and paternal dwelling time in Guiyu were negatively correlated with TSH. Age was negatively correlated with IGFBP-3. Multiple stepwise regression analysis was used to evaluate factors related to each hormone in children from Guiyu (Table 6). FT4 was positively associated with FT3, while fever was negatively associated with FT3. Vitamin consumption, suffered from any disease, duration of outdoor play, and house functions as a workshop were negatively associated with FT4. Father's work involved in e-waste recycling activities was positively associated with FT4. Eating bean products, suffered from any disease, paternal dwelling time in Guiyu, and working hours of the mother were negatively associated with TSH. Residence of child was positively associated with TSH. IGFBP-3 and fevers were positively associated with IGF-1. House close to an e-waste site was positively associated with IGFBP-3.

**Table 5:** Spearman correlation coefficients between hormones and some investigated factors in children, Guiyu, China, 2010.

Related factors	FT3(n = 162)		FT4(n = 162)		TSH(n = 162)		IGF-1(n = 162)		IGFBP-3(n = 162)	
	*r_s_*	*p*	*r_s_*	*p*	*r_s_*	*p*	*r_s_*	*p*	*r_s_*	*p*
Gender	0.016	0.838	−0.162	0.039	0.000	0.997	−0.079	0.317	−0.073	0.358
Age (years)	−0.034	0.709	−0.025	0.787	0.012	0.899	0.003	0.976	−0.196	0.030
BMI	0.170	0.033	−0.019	0.809	0.121	0.130	−0.067	0.401	−0.028	0.725
The habit of biting fingers	−0.057	0.507	−0.078	0.365	−0.207	0.015	−0.011	0.894	0.011	0.903
The frequency of eating bean products	−0.026	0.759	−0.034	0.697	−0.182	0.033	0.030	0.727	−0.101	0.238
The frequency of taking vitamin	0.014	0.871	−0.219	0.010	0.141	0.100	−0.132	0.126	−0.008	0.923
Whether suffered from any disease	−0.074	0.390	−0.188	0.028	−0.146	0.089	0.104	0.227	0.025	0.769
Household drinking water sources	−0.049	0.569	−0.181	0.035	0.024	0.782	−0.019	0.829	0.008	0.927
Residence of the father	−0.037	0.668	−0.088	0.305	−0.196	0.022	−0.100	0.246	−0.029	0.736
Working hours of the mother	−0.086	0.318	−0.188	0.028	−0.148	0.085	0.006	0.949	0.117	0.173
House functioned as workshop	−0.068	0.430	−0.217	0.011	0.096	0.262	−0.068	0.431	−0.020	0.813

**Table 6:** Multiple stepwise regression analysis between PBDEs, Lead, Cadmium, FT3, FT4, TSH, IGP-1, IGPBP-3 and related factors in children, Guiyu, China, 2010.

Related factors	*B*	β	*Partial correlation*	*R^2^*	*Adjusted R^2^*	*F*	*p*	*95% CI for* β
BDE28 Models				0.125	0.106	6.355		
TSH	3.776	0.260	0.258				0.003	1.349–6.203
The habit of biting fingers	3.623	0.179	0.181				0.036	0.240–7.006
The frequency of eating bean products	−4.624	−0.202	−0.218				0.017	−8.413—0.835
BDE47 Models				0.127	0.100	4.781		
TSH	4.698	0.217	0.220				0.011	1.106–8.289
IGFBP-3	−0.131	−0.165	−0.174				0.045	−0.258—0.003
The habit of biting fingers	5.657	0.187	0.191				0.027	0.660–10.654
Father's education levels	6.164	0.196	0.204				0.018	1.079–11.250
BDE100 Models				0.035	0.028	4.884		
The frequency of eating bean products	−1.549	−0.187	−0.187				0.029	−2.935—0.163
BDE99 Models				0.181	0.150	5.804		
TSH	2.429	0.236	0.252				0.003	0.814–4.044
FT3	−1.776	−0.243	−0.251				0.004	−2.957—0.594
Gender	−4.252	−0.196	−0.208				0.016	−7.706—0.797
Fever	−1.648	−0.175	−0.184				0.034	−3.170—0.125
Whether decorate house	−3.573	−0.165	−0.176				0.042	−7.023—0.123
BDE154 Models				0.102	0.089	7.611		
The frequency of eating dairy	−1.705	−0.264	−0.268				0.002	−2.753—0.657
The habit of dietary choosiness	2.251	0.185	0.191				0.026	0.278–4.225
BDE153 Models				0.037	0.030	5.168		
Lead	0.512	0.192	0.192				0.025	0.067–0.958
BDE209 Models				0.102	0.081	5.012		
TSH	18.432	0.185	0.191				0.027	2.157–34.708
FT3	−13.054	−0.184	−0.190				0.027	−24.601—1.506
The frequency of eating dairy	25.104	0.221	0.226				0.008	6.536–43.671
Total PBDEs Models				0.126	0.106	6.400		
TSH	31.713	0.273	0.279				0.001	13.002–50.425
FT3	−15.986	−0.194	−0.202				0.019	−29.262—2.709
The frequency of eating dairy	23.629	0.179	0.187				0.030	2.282–44.976
Lead Models				0.104	0.084	5.158		
Age	1.239	0.172	0.177				0.040	0.057–2.421
Residence of the mother	0.993	0.213	0.218				0.011	0.232–1.754
House close to e-waste	2.104	0.214	0.218				0.011	0.486–3.722
Cadmium Models				0.176	0.151	7.049		
FT3	−0.032	−0.167	−0.180				0.038	−0.063—0.002
Head circumference	0.050	0.254	0.268				0.002	0.019–0.081
Mother's education levels	−0.080	−0.219	−0.230				0.008	−0.138—0.022
Whether suffered from any disease	−0.224	−0.221	−0.231				0.007	−0.387—0.061
FT3 Models				0.162	0.150	12.982		
FT4	0.101	0.331	0.339				0.000	0.053–0.148
Fever	−0.266	−0.206	−0.219				0.010	−0.468—0.064
FT4 Models				0.296	0.263	9.097		
FT3	1.140	0.347	0.378				0.000	0.655–1.624
Whether suffered from any disease	−3.184	−0.185	−0.212				0.015	−5.736—0.632
The frequency of taking vitamin	−1.613	−0.274	−0.303				0.000	−2.494—0.732
The length of outdoor play	−0.833	−0.150	−0.173				0.048	−1.658—0.008
Father's work involved in e-waste recycling	3.121	0.231	0.250				0.004	1.024—5.219
House functions as workshop	−2.268	−0.232	−0.255				0.003	−3.763—0.774
TSH Models				0.267	0.233	7.898		
FT4	−0.054	−0.251	−0.269				0.022	1.023–1.334
The frequency of eating bean products	−0.308	−0.196	−0.222				0.017	1.019–1.212
Whether suffered from any disease	−0.780	−0.209	−0.232				0.030	0.002–0.724
Residence of the father	−0.539	−0.373	−0.354				0.023	0.688–0.972
Residence of the child	0.347	0.244	0.240				0.023	1.043–1.743
Working hours of the mother	−0.198	−0.249	−0.264				0.000	1.280–2.271
IGF-1 Models				0.115	0.102	8.699		
IGFBP-3	1.803	0.199	0.205				0.017	0.331–3.276
Fever	56.353	0.248	0.252				0.003	19.445–93.261
IGFBP-3 Models				0.087	0.073	6.349		
IGF-1	0.028	0.250	0.252				0.003	0.010–0.046
House close to an e-waste site	10.329	0.179	0.184				0.032	0.882–19.777

Multiple stepwise regression analysis was also used to evaluate factors related to blood lead and cadmium in children from Guiyu (Table 6). Age, residence of mother, and house close to an e-waste recycling site were positively associated with blood lead. Head circumference was positively associated with blood cadmium. FT3, mother's education level, and children suffered from any disease had a negative association with blood cadmium.

## Discussion

We demonstrate that serum total PBDEs geometric mean (GM) was 162.98 ng/g lipid and ranged from 16.56 to 582.35 ng/g lipid in children residing in Guiyu. Before this work, a small study compared PBDE levels in children from another e-waste recycling area and a reference site in Taizhou, Zhejiang province, China [36]. The sum of PBDEs reported here in Guiyu children was much higher than that in Taizhou except BDE209, which may indirectly reflect more serious environmental pollution resulting from disassembling of electronic devices in Guiyu [37]. Although Guiyu and Taizhou are two famous e-waste destinations in China, the e-waste disassembling method is not the same. Most workshops in Guiyu are small family run, home-based workshops using rudimentary and informal procedures that pose more serious health problems to the environment and humans compared to those in Taizhou. “The Handbook of Environmental Chemistry” identifies Guiyu as the largest and the second most polluted site in the world due to its informal recycling processes (acid extraction for metals, open burning of wires to get copper) [38]. Decreases in PBDE concentrations are observed with increasing distance from workshops in samples linked with acid processing of waste [39]. Similar results are found when family residences serve as workshops. One of our previous studies indicates that the PBDE concentration of surface soil samples in residential areas is much higher than that in agricultural areas in Guiyu, suggesting that leakage of PBDEs into the environment occurs from informal e-waste recycling [5]. Therefore, more attention should be given to the health of people living in Guiyu, especially for children residing near workshops or house used as a workshop.

In this study, BDE209 is the predominant congener among the eight BDEs tested, with a range from ND to 470.29 ng/g lipid, in spite of the detection frequency is 71.72%, which may be attributed to its large molecular weight, resulting in relatively rapid excretion after biotransformation [40]. Another study conducted among female e-waste recycling workers in Guiyu found that BDE209 can be as high as 1640 ng/g lipid, with a median of 83.5 ng/g lipid [41]. The half-life of BDE209 is only 15 days in human [42], and BDE209 concentration in child serum show high values, on one hand indicating that PBDEs exposure in Guiyu is high and continuous, and also suggesting a broad use of BDE209 in industry [43]. On the other hand implying the degradation rate of BDE209 in the human body is slower than its intake rate [44]. A top-level predator tends to have a high uptake and accumulation of higher bromine compounds [45]. BDE209 is rich in plasma and blood-rich tissues due to the high plasma protein-binding properties of deca-BDE [46]. Consistent with our previous study, prior investigations find that BDE209 is present in high concentrations in the environmental matrix of Guiyu, including air, road and farmland soil, and e-waste dumpsite soil [5], [7], [47], [48]. In spite of BDE209 being the major PBDE in children, it does not indicate that BDE209 is the most influential on thyroid function [49]. In this study, BDE209 is weakly associated with TSH and IGFBP-3. Further studies are suggested.

When compared with children from around the world, PBDE concentrations in the present study were higher than those in newborns and 4-year-old children from Menorca Island [50], 0–11-year-old children from Dalian, China [51], 6–13-year-old children from Mexico [52], newborns from Indiana, USA [53], 0–15-year-old children from Australia [54], and 7-year-old children from Faroe Island [55], and comparable with those in California Mexican-American children, USA [56], but lower than those in the children residing in Berkeley, California USA [57]. All together, these results are in concordance with those reported in other studies, where people in Asia are exposed to comparable or lower levels than people living in North American and much higher than people in Europe. In addition, several studies showed that PBDE concentrations in children were higher than those of the mothers [54], [56], [58].

To our knowledge, this is thus far the first study to include lead and cadmium as co-exposures when investigating associations between PBDEs and key growth- and development-related hormones in children from an e-waste area. One prior study investigated the relationship between metals in blood and urine, and thyroid function among adults in the United States, and the authors found blood cadmium levels to be inversely related with the logarithm of TSH, and blood lead levels was correlated with decreased T4 [59]. However, no relationships are observed between lead, cadmium and thyroid or growth-related hormones among children in this study. We suggest further study should be performed on the relationships and mechanisms between heavy metals and thyroid and growth hormones.

This study finds out that when the house functions as an e-waste recycling workshop negatively associate with FT4 of children, suggests that living in an e-waste dismantling area or in a family-run informal e-waste workshop might cause changes in thyroid hormones, a conclusion differing from prior studies. Exposure to brominated flame retardants released from informal e-waste handling might result in changes in THs and TSH levels [60], whereas in that study, the TSH levels in the e-waste recycling occupational-exposed groups were lower than that in the non-occupational-exposed group. These results are consistent with several studies that found PBDEs were negatively associated with TSH [61]–[63]. However, the TSH was positively correlated with both BDE154 and total PBDE in this study. According to the study conducted by Han, who also found PBDEs were negatively associated with TSH during low concentration range (<3 µIU/mL), whereas positively associated with TSH within high concentration range (≥3 µIU/mL) [64]. Exhilaratingly, the TSH levels (Mean±SD, 2.85±1.09) in this study generally fall in the high concentration range, which is consistent with the result from Han [64]. The studies show a negative association between PBDEs and TSH all with low concentration of TSH [62], [65]. This may be because the PBDE congeners were different, and the participants in our study were all from an e-waste recycling region. Children seemed to be particularly appropriate for a monitoring program, as they are not directly exposed to occupational pollution; thus, children normally reflect present trends of environmental exposure more accurately than adults [66]. In addition, serum TSH concentrations may vary with age, and several epidemiological and animal studies find that association between TSH levels and PBDE exposure maintain inconsistency [67]–[73]. Therefore, it is difficult to make direct comparisons between occupational-exposed populations or the prenatal-exposed population in the present study. Positive associations between BDE209 and IGFBP-3 are observed in this study. Another study demonstrated that the log IGF-1 level is significantly linked to an increase in the log BDE196 level and a decrease in the log BDE85 level [74]. However, we did not find any associations between IGF-1 and any of the eight PBDE congeners tested or the total PBDE.

One limitation is that we did not obtain other organic pollutant such as polychlorinated biphenyls (PCBs), another important endocrine disruptor, due to insufficient blood. However, the outcomes of our study still strongly suggest that elevated concentrations of PBDEs may cause adverse effects on child hormone function. Thyroid hormones affect the function of nearly all tissues via their effects on cellular metabolism and play an essential role in differentiation and growth. Interference with thyroid hormone homeostasis by environmental compounds has the potential to impact development [75]. Obviously, there are a variety of chemical contaminants in e-waste and we cannot measure all potentially relevant toxicants, but our results included other potentially relevant chemicals (lead and cadmium) that are present in informal e-waste recycling areas. Another limitation is that the present study was conducted in a cross-sectional design that may raise an issue of validity of causal inferences between exposures and hormone alterations. However, our study can serve as a reference for further studies performed at similar sites and in similar populations, or serve as a foundation for policy makers.

In conclusion, the present study supports the finding that elevated serum PBDEs are associated with thyroid hormone alterations, and that elevated blood lead levels are common in children at an e-waste area. BDE153 is positively correlated with blood lead levels in children from Guiyu. More research is needed to differentiate mechanisms governing TH levels between e-waste recycling sites and non-e-waste-recycling sites. Our findings support efforts of the EU RoHS Directive to restrict the use of PBDEs, lead and other hazardous substances in electronic equipment.

## Supporting Information

Table S1 **Spearman correlation analysis among PBDEs (ng/g lipids), Heavy Metals, Thyroid Hormone and Growth Hormone in Serum of Children, Guiyu, China, 2010.**
(DOCX)
