# Supplementary material for: Elevated Serum Polybrominated Diphenyl Ethers and Alteration of Thyroid Hormones in Children from Guiyu, China
Source: PLoS One. 2014 Nov 21;9(11):e113699. doi: 10.1371/journal.pone.0113699 (PMC4240651; doi:10.1371/journal.pone.0113699)
Supplement: Table S1 — Spearman correlation analysis among PBDEs (ng/g lipids), Heavy Metals, Thyroid Hormone and Growth Hormone in Serum of Children, Guiyu, China, 2010. (DOCX) [file pone.0113699.s001.docx]

**Table S1.** Spearman correlation analysis among PBDEs (ng/g lipids), Heavy Metals, Thyroid Hormone and Growth Hormone in Serum of Children, Guiyu, China, 2010.

|  | **BDE28** | **BDE47** | **BDE100** | **BDE99** | **BDE154** | **BDE153** | **BDE183** | **BDE209** | **∑PBDE** | **Lead** | **Cadmium** | **fT3** | **fT4** | **TSH** | **IGF-1** | **IGFBP-3** |
| --- | --- | --- | --- | --- | --- | --- | --- | --- | --- | --- | --- | --- | --- | --- | --- | --- |
| BDE28 | 1.000 |  |  |  |  |  |  |  |  |  |  |  |  |  |  |  |
| BDE47 | 0.852^**^ | 1.000 |  |  |  |  |  |  |  |  |  |  |  |  |  |  |
| BDE100 | 0.654^**^ | 0.626^**^ | 1.000 |  |  |  |  |  |  |  |  |  |  |  |  |  |
| BDE99 | 0.722^**^ | 0.787^**^ | 0.820^**^ | 1.000 |  |  |  |  |  |  |  |  |  |  |  |  |
| BDE154 | 0.338^**^ | 0.270^**^ | 0.703^**^ | 0.526^**^ | 1.000 |  |  |  |  |  |  |  |  |  |  |  |
| BDE153 | 0.174 | 0.166 | 0.360^**^ | 0.294^**^ | 0.627^**^ | 1.000 |  |  |  |  |  |  |  |  |  |  |
| BDE183 | 0.159 | 0.174 | 0.465^**^ | 0.343^**^ | 0.574^**^ | 0.715^**^ | 1.000 |  |  |  |  |  |  |  |  |  |
| BDE209 | 0.319^**^ | 0.213^**^ | 0.797^**^ | 0.478^**^ | 0.750^**^ | 0.286^**^ | 0.475^**^ | 1.000 |  |  |  |  |  |  |  |  |
| ∑PBDE | 0.618^**^ | 0.553^**^ | 0.921^**^ | 0.728^**^ | 0.794^**^ | 0.482^**^ | 0.598^**^ | 0.891^**^ | 1.000 |  |  |  |  |  |  |  |
| Lead | -0.008 | -0.065 | 0.039 | -0.070 | 0.142 | 0.186^*^ | 0.188^*^ | -0.083 | -0.090 | 1.000 |  |  |  |  |  |  |
| Cadmium | 0.040 | 0.077 | 0.079 | 0.033 | 0.085 | 0.114 | 0.122 | 0.100 | 0.034 | 0.186 | 1.000 |  |  |  |  |  |
| fT3 | 0.015 | 0.061 | -0.082^*^ | -0.054 | -0.174 | 0.046 | -0.055 | -0.188 | -0.123 | -0.001 | -0.203 | 1.000 |  |  |  |  |
| fT4 | -0.025 | -0.064 | -0.219^*^ | -0.121 | -0.243^*^ | -0.224^*^ | -0.244^*^ | -0.194 | -0.202 | -0.005 | -0.068 | 0.213^**^ | 1.000 |  |  |  |
| TSH | 0.327^**^ | 0.269^**^ | 0.348^**^ | 0.299^**^ | 0.320^**^ | 0.193 | 0.191 | 0.205^*^ | 0.326^**^ | -0.051 | 0.099 | 0.044 | -0.159^**^ | 1.000 |  |  |
| IGF1 | -0.073 | -0.130 | -0.040 | -0.119 | -0.013 | -0.001 | 0.119 | 0.025 | -0.009 | -0.023 | -0.023 | 0.007 | 0.102 | 0.009 | 1.000 |  |
| IGFBP3 | -0.011 | -0.095 | 0.138 | -0.008 | 0.129 | 0.095 | 0.125 | 0.247^*^ | 0.172 | -0.015 | -0.080 | -0.087 | -0.056 | -0.004 | 0.295^**^ | 1.000 |

Abbreviations: CI, confidence interval; BDE, brominated diphenyl ether; FT3, free triiodothyronine; FT4, free thyroxine; IGF-1, insulin-like growth factor 1; IGFBP-3, insulin-like growth factor binding protein 3; ND, not detectable; TSH, thyroid-stimulating hormone.

^*^*P*<0.05, ^**^*P*<0.01
